# Disruption of Skin Stem Cell Homeostasis following Transplacental Arsenicosis; Alleviation by Combined Intake of Selenium and Curcumin

**DOI:** 10.1371/journal.pone.0142818

**Published:** 2015-12-01

**Authors:** Shiv Poojan, Sushil Kumar, Vikas Verma, Anupam Dhasmana, Mohtashim Lohani, Mukesh K. Verma

**Affiliations:** 1 Environmental Carcinogenesis Division, CSIR-Indian Institute of Toxicology Research, Mahatma Gandhi Marg, P Box 80, Lucknow-226001, India; 2 Environmental Carcinogenesis & Toxicoinformatics Laboratory, Department of Bioengineering, Integral University, Lucknow-226026, India; 3 Environmental Carcinogenesis & Toxicoinformatics Laboratory, Department of Biosciences, Integral University, Lucknow-226026, India; University of Kentucky, UNITED STATES

## Abstract

Of late, a consirable interest has grown in literature on early development of arsenicosis and untimely death in humans after exposure to iAs in drinking water *in utero* or during the childhood. The mechanism of this kind of intrauterine arsenic poisoning is not known; however it is often suggested to involve stem cells. We looked into this possibility by investigating in mice the influence of chronic *in utero* exposure to arsenical drinking water preliminarily on multipotent adult stem cell and progenitor cell counts at the beginning of neonatal age. We found that repeated intake of 42.5 or 85ppm iAs in drinking water by pregnant BALB/c mice substantially changed the counts of EpASCs, the progenitor cells, and the differentiated cells in epidermis of their zero day old neonates. EpASCs counts decreased considerably and the differentiated / apoptosed cell counts increased extensively whereas the counts of progenitor cell displayed a biphasic effect. The observed trend of response was dose-dependent and statistically significant. These observations signified a disruption in stem cell homeostasis. The disorder was in parallel with changes in expression of biomarkers of stem cell and progenitor (TA) cell besides changes in expression of pro-inflammatory and antioxidant molecules namely Nrf2, NFkB, TNF-α, and GSH. The biological monitoring of exposure to iAs and the ensuing transplacental toxicity was verifiable correspondingly by the increase in iAs burden in hair, kidney, skin, liver of nulliparous female mice and the onset of chromosomal aberrations in neonate bone marrow cells. The combined intake of selenite and curcumin *in utero* was found to prevent the disruption of homeostasis and associated biochemical changes to a great extent. The mechanism of prevention seemed possibly to involve (a) curcumin and Keap-1 interaction, (b) consequent escalated de novo GSH biosynthesis, and (c) the resultant toxicant disposition. These observations are important with respect to the development of vulnerability to arsenicosis and other morbidities later in life after repeated *in utero* or postnatal exposure to iAs in drinking water that may occur speculatively through impairment of adult stem cell dependent innate tissue repair mechanism.

**Highlights**

- Chronic exposure to arsenite *in utero* disrupted adult stem cell homeostasis.
- Counts of adult stem cell and progenitor cell changed in neonatal mouse epidermis.
- Levels of stem cell and differentiated cell markers modulated correspondingly.
- TNF, Nrf2, NFkB, GSH, and tissue iAs load modulation were key events.
- *In utero* exposure to a combination of selenite and curcumin mitigated these effects.

## Introduction

Of late, a considerable interest has grown in literature on early development of arsenicosis as well as untimely mortality later in human life after repeated exposure to arsenical drinking water in utero and during the childhood. The underlying mechanism is not known; however, stem cells are speculated to be involved. It is based on the premise that, like somatic cells, the multipotent adult stem cells may also get affected during chronic intrauterine exposure to Inorganic arsenic (iAs).

iAs is a multisite transplacental toxicant, carcinogen, as well as deliberate homicidal toxicant [1–10]. The clinical manifestations of arsenic poisoning in humans include stillbirth, infant-deaths, impairment in children’s lung and intellectual function, neuro-toxicity, increased cancer incidence in adults and an increased mortality from cancer and bronchiectasis in adolescents [11–15].

In embryology, it is understood that after establishment of the germ cell layers in embryogenesis, an optimal pool of adult stem cells and progenitor cell count is apportioned for normal organogenesis. The optimum numbers are considered to be necessary for the tissue growth during embryogenesis as well as in wound repair both in utero and in postnatal stages [16]. In view of this tacit information, it is reasonable to believe that the manipulation of stem cell numbers by chronic exposure to iAs in utero or in postnatal age could disorder the optimal dynamics of EpASC homeostasis in tissues and eventually the organ growth. This plausibility however has seldom been explored [17–20].

In current study, we have investigated this apparently valid issue using an enriched population of adult stem cell isolated from neonate mouse skin i.e. the EpASCs. Lesions in skin are the hallmarks of iAs toxicity observed after chronic exposure to arsenic-contaminated drinking water [1]; hence, EpASCs from epidermis have been deployed for test of hypothesis. The cultured mouse putative epidermal stem cells are proposed as a potential tool to study stem cell biology [21]. We have investigated experimentally the potential of chronic intrauterine iAs exposure to manipulate EpASCs pool size in zero day old neonate mouse skin; we determined the manipulating potential by measuring alterations in EpASCs counts in the tissue. To characterize iAs toxicity in adult stem cell, we have studied changes in levels of oxidative stress and inflammation molecular mediators and also the changes in expression levels of stem cell and differentiated cell biomarkers ex vivo. For biological monitoring of transplacental iAs toxicity, status of chromosomal aberrations has been determined in bone marrow cells of neonates exposed to arsenical drinking water in utero. This study is an in-utero repeat dose toxicity study and not a long term follow-up.

We have also attempted the chemoprevention of toxic effects in stem cells using essential micronutrient selenite and the food additive curcumin both in vivo and in vitro [20, 22]. Possible interaction of selenite and curcumin with key regulators of oxidative stress and inflammation and their potential to regenerate antioxidant GSH has been evaluated using in silico studies.

## Materials and Methods

### The *in vitro* studies

#### EpASCs culture and dose selection

The multipotent adult stem cells were isolated from epidermis of neonate BALB/c mouse skin, and were cultured as described earlier [23,24]. In brief, the excised epidermis was digested enzymatically; keratinocytes were isolated and cultured in growth-promoting medium in a CO_2_ incubator set at 5% CO_2_ and 37°C. Putative stem cells were seeded in collagen-fibronectin pre-coated flasks and placed in CO_2_ incubator for 10 min to allow adhering [25]. Culture medium was first changed after 4 days and thereafter on alternate days to culture EpASCs. The EC_50_ value of arsenite, selenite, and curcumin in EpASCs was determined using MTT-based cell viability assay.

#### EC_50_ determination

EpASCs from neonates born to mothers in control group were employed for the assay. Cells were seeded (2*10^4^ cells/well) in 96-well plates and allowed to adhere for 24 hours in a CO_2_ incubator. iAs and selenite were dissolved in MilliQ water; curcumin was dissolved in DMSO. Final concentration of DMSO in the assay was ≤0.05%; flasks from the control group received only the vehicle. Cells were exposed (in triplicate, for 24 hours) to serial dilutions (40 to 1.25 μM) of arsenite (NaAsO_2_, Sigma catalogue #S7400) or selenite (Na_2_SeO_3_, Sigma catalogue #S5261) or curcumin (Sigma catalogue #C1386). After 24h exposure, MTT (10μl of 5mg/ml) was added and cells were further incubated for 3-4hrs. The formazan crystals formed in viable cells were dissolved in DMSO. A_540_ was determined using a microplate reader (Microquant, Bio-Tek, USA) to establish EC_50_ value of arsenite, selenite and curcumin (S1a Fig).

#### CMFDA-reactive GSH

Changes in intracellular level of GSH were studied in EpASCs using CMFDA (Molecular Probes, Eugene OR USA). EpASCs were seeded on chamber slides. After exposure to test items, cells were incubated in CO_2_ incubator with pre-warmed (37°C) 10μM CMFDA (prepared in culture medium). Following incubation for 30 min, CMFDA-media was replaced with culture medium without CMFDA (fresh and pre-warmed). The cells were incubated additionally for 30 min and washed with PBS to fix subsequently in 3.7% paraformaldehyde. CMFDA-stained cells were viewed under fluorescence microscope and photographed for documentation.

#### Immunoassay

EpASCs proteins were extracted by sonication using the Lysis buffer (Sigma-Aldrich) containing a cocktail of protease and phosphatase inhibitors. Cell-lysates were centrifuged (13,000g, 4°C, 15 min) and the supernatant was saved. The protein concentration was determined by the Bradford method. Aliquots (40 μg protein) from each sample were first heat-inactivated (95°C, 10 min) in denaturing buffer [10% glycerol, 1% SDS, 1% β-mercapto-ethanol, 0.01% bromophenol blue, 10 mMTris-HCl (pH 6.8)], and then electrophoresed on 12% polyacrylamide gel. Protein bands were blotted onto Immobilon-P (Millipore) already pre-blocked for unspecific protein binding (1 hour with 5% skimmed milk in TBS-T buffer, pH 7.6). Blots were washed extensively three times in 0.1% Tween-20 in TBS for subsequent incubation with primary antibodies (overnight, 4°C). The primary antibody for Nrf2 (Sigma), NFkB (Invitrogen), CK10, CK14, (Santa Cruz Biotechnology), PCNA, P38, p63, TNF-α (Cell Signaling)was used in 1:5,000 dilution and the HRP conjugated secondary antibody (Cell Signaling) was used in 1:10,000 dilution. The probed membranes were incubated with substrate (5 min, RT) and developed with Enhanced Chemiluminescence (ECL) kit (Thermo, USA). Immunoblots were stripped and re-probed with β-actin antibody (Sigma-Aldrich) for loading correction.

### The *in vivo* studies

#### Animals and treatment

Pregnant BALB/c mice (20-25g) carrying 6-day old embryos were employed in the study. Animals were randomized into seven groups of five pregnant mice in each, and housed at 25°C with 12-h light-dark cycle period. For randomization, the random numbers were allotted to animals for the treatment conditions from the random number table.

Mice were fed arsenic-free standard pellet diet and safe drinking water ad lib (Group 1). The experimental group of mice received sodium arsenite (42.5ppm or 85ppm) in drinking water (i.e. arsenical drinking water) ad libitum during the gestation period of 8–18 days (Group 2 & 3). Doses showing transplacental carcinogenic potential in mice were selected from the study of Waalkeset al [25] so as to ensure the in utero toxicity. For chemoprevention of toxicity, sodium selenite (2.8 or 5.6 mg/kg b wt [26] or curcumin (50 or 100mg/kg b wt [27] were aseptically administered by oral gavage in a total volume of 0.2 ml either alone in parallel to iAs exposure (Group 4 & 5) or in combination (Group 6 & 7); curcumin was mixed homogeneously with an aqueous solution of gum acacia. Control animals received n-saline in place of chemopreventive test items.

After iAs exposures, the zero day old neonates were processed as earlier for stem cell isolation and FACS based characterization [21, 23–24]. All experiments were approved by the CSIR-IITR IAEC (Institutional Animal Ethics Committee) according to CPCSEA (Committee for the Purpose of Control and Supervision of Experiments on Animals) Guidelines of Government of India.

#### EpASCs labeling and FACS based characterization

BrdU (50mg/ kg b. wt., Sigma catalogue #B5002) was administered to female mice twice a day for four days before mating. The LRKs were examined in neonatal epidermis keratinocyte isolates after a chase period of 28 days [24]. After excising skin and stripping off epidermis, EpASCs were isolated and cultured ex vivo as described before. Before use, cells were washed twice with cold PBS. One million cells were transferred into a polystyrene tube and pelleted. After washing with 0.5% Tween-20, the pelleted cells were mixed with 10 ml FITC-conjugated anti-BrdU antibody. Contents were vortexed and left at RT for 30 min. Cells were finally washed again twice with PBS and resuspended in 1 ml PBS before BrdU label determination using a BD-FACS-LSR II flowcytometer. Neonatal keratinocytes from the mice of control or the experimental group were pooled before analyses, and levels of BrdU label determined in triplicate.

#### Chromosomal aberration in bone marrow cells

Neonates (0 day old), borne to the mother mice receiving treatment as described above (six groups in total), were sacrificed. Group 1 (control) received normal drinking water; Groups 2 to 6 received arsenite (85ppm) in drinking water and the additives. The selenite dose formulation (5.6 mg/kg b wt) was prepared in distilled water and curcumin (100mg/kg b wt, PO) in aqueous suspension of gum acacia. Only clear solutions of selenite and arsenite or homogeneous suspensions of curcumin were administered by oral gavage; and dose of each test item was administered in a total volume of 0.2 ml (per day) for 30 days. Animals in control group were administered n-saline in place of chemopreventive test items.

Each neonate mouse was injected with 0.04% colchicine 1 mg/100g b wt. i.p. (BW, Sigma, USA) ninty minutes prior to the sacrifice. Femurs were excised and skeletal muscles were stripped off; the bones were crushed to collect marrow cells in 75mM KCl (hypotonic solution). After incubation for 20 min at 37°C, cells from 6 pups were pooled and fixed in 3:1 methanol-glacial acetic acid. Chromosome preparations were made using the standard procedure of air drying, and were stained with 7% Giemsa solution (Merck, India). Slides were coded and blind-scored.

A set of 300 cells was examined in each group. Normal cells showed clear metaphase with normal chromosomes. The aberration types were identified as per the standard guidelines for evaluation of genetic toxicity. Cells with one or more aberrations were counted and scored as a percent of cells with CA with respect to control group. Number of CB, CG, RF and CF were listed as percentages of total % CA. Data are presented as mean ± standard deviation.

#### Tissue iAs load determination

Briefly, adult nulliparous female mice (20g b wt) were distributed into 6 groups of 10 animals in each as described above in the chromosomal aberration study and exposed to iAs in drinking water for 30 days. Hair, liver, kidney, and skin specimens were excised from control and experimental group of mice; and were stored frozen in acid-free vials in liquid nitrogen until analysis. For analyses, the specimens were thawed and 100 mg tissue samples were acid-digested with 2 ml concentrated nitric acid using a microwave oven. The mineralized contents were made up to 10 ml using de-ionized water. iAs content was determined using an Atomic Absorption Spectrophotometer (AAnalyst 300, Perkin Elmer, USA) equipped with a Flow Injection system (FIAS-100). The iAs was determined using a four-point standard curve prepared from a standard reference solution for arsenic, and quality control standards were run to calibrate the AAS.

### The *in silico* study

It was performed to explore possibility of curcumin, or selenite influencing the Nrf2-Keap1 dependent antioxidant regeneration pathway. Docking of ligand Keap-1 with curcumin or GS-AsH-SG or GS-Se-SG (S2a–S2c Fig) was simulated using PatchDock. The operation of protein-protein docking of Nrf2 with natural ligand Keap-1 and with Keap1-Curcumin complex was also simulated and compared using ZDOCK v2.5 module of Discovery Studio.

#### Data Analysis and Statistics

All the in vitro and ex vivo studies were performed in triplicate and were repeated three times. Data are average of three mean values. The data from in vivo studies represent an average of five pregnant (n = 5) or ten nulliparous mice (n = 10). The results were analyzed statistically using one-way analysis of variance control (ANOVA-non parametric) using the Prism Graph Pad software; ‘p’ values <0.05 were considered as significant [28]. Student’s t-test [29] was also applied to compare the results of experimental group with the control group. Bone marrow cell recovery was expressed as the mean number of bone marrow cells obtained from a pool of a neonate’s femurs.

## Results

### FACS based analyses of LRK pool size

**Fig 1:**
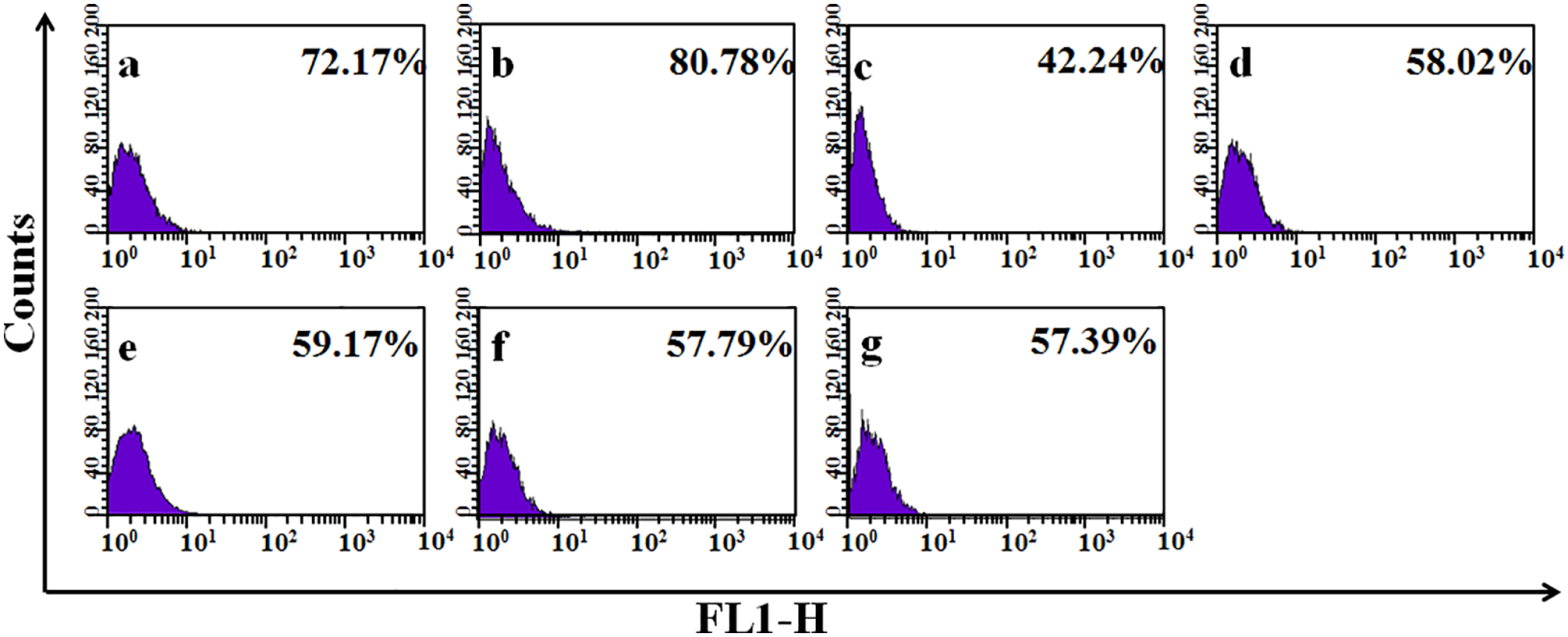
Yields of LRK in neonate epidermis after *in utero* exposure to iAs and the additives. (A) control, (B) 42.5ppm iAs, (C) 85ppm iAs, (D) 85ppm iAs + selenite (5.6mg mg/kg b wt), (E) 85ppm iAs + curcumin (100mg/kg b wt), (F) 85ppm iAs + selenite + curcumin, (G) 85ppm iAs + ½ dose (selenite + curcumin); Note: exhibit is a FACS generated sketch of cell counts vs. amounts of retained BrdU label displaying unique dispersion patterns of LRK counts. It was visible in framework of the relative fluorescence value outlined on y- axis and the keratinocytes count showing the respective FL1-H counts of fluorescent BrdU-LRKs charted on x-axis. Cells with lowest concentration of BrdU were found to be large in counts, and cells with highest concentration of the fluorescent label were found to be less in counts. The latter group of cells represented the stem cell population in LRK isolates [16, 57]. LRKs were a heterogeneous population of cells as these were isolated from a cluster of neonates born to a group of mother mice.

The profile of EpASCs pool size in the neonate epidermis is displayed in Fig 1. In the control group, the total yield of LRKs was found to be 72.17% with a unique dispersion pattern. The pattern and total yield of LRKs changed remarkably in the group receiving chronic in utero exposure to iAs in drinking water (Fig 1B–1G). At 42.5ppm iAs dose (Fig 1B), total yield of LRKs increased to 80.78% whereas at 85ppm iAs dose, the yield decreased to 42.24% (Fig 1C). In 42.5ppm iAs exposure group, the marginal increase in LRKs yield over the control value accompanied with a distinctive change in dispersion pattern. In 85ppm iAs exposure group, the significant loss (>40%) portrayed a radical change in dispersion pattern. The observed trend in response was dose dependent.

**Fig 2:**
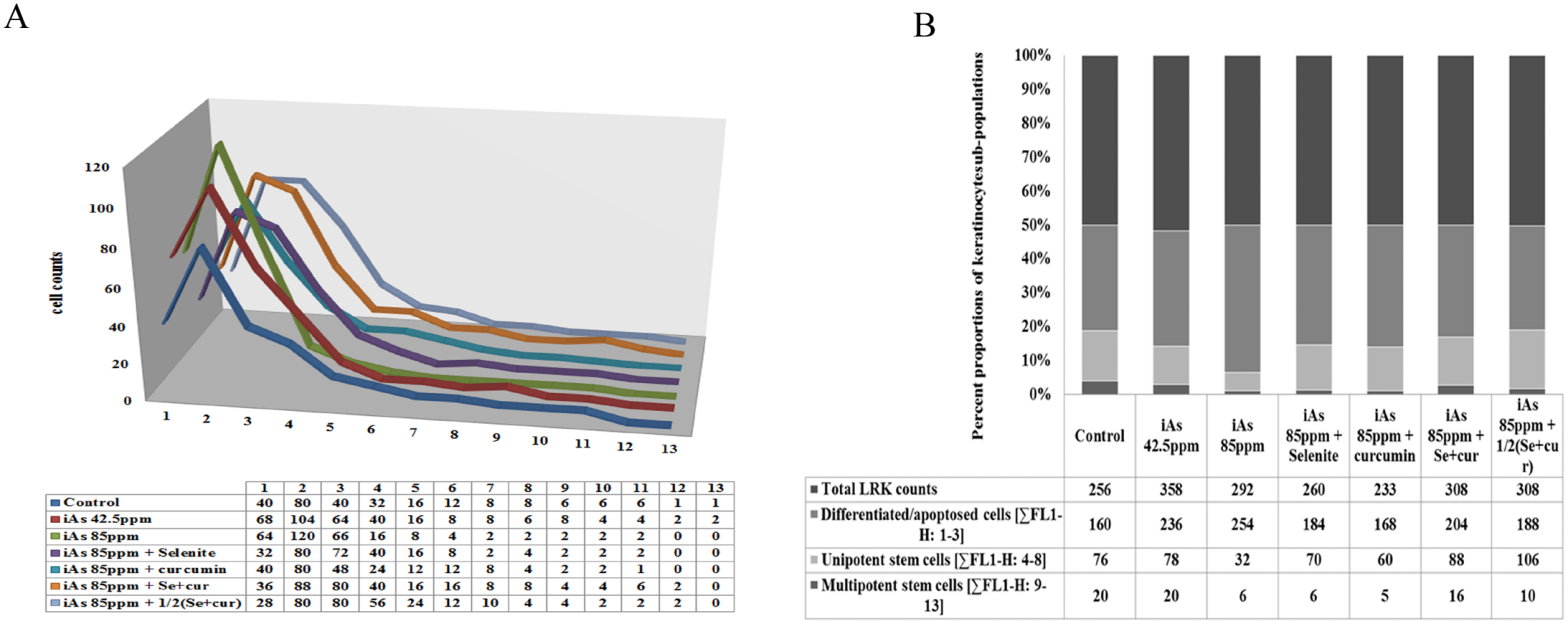
EpASCs, TA, and differentiated cell count profile in LRK pool from neonate epidermis after *in utero* exposures to iAs and the additives. (A) the change in cell counts per 1/10^th^ change in FL1-H value were plotted; numerical values represent maximum cell counts per 1/10^th^ of change in label density shown in Fig 1A–1G; (B) the cell counts showing approximately similar degree of change [∑ FL1-H 1–3, ∑ FL1-H 4–8, ∑ FL1-H 9–13] showcased three sub-populations of cells [16, 57] namely keratinocytes retaining (i) greatest quantity of BrdU label i.e. adult stem cells (EpASCs), (ii) least amount of BrdUlabel i.e. differentiated or apoptosed cells, and (iii) intermediate amount of BrdU label i.e. the progenitor TA cells on the way to differentiation.

This inconsistency in LRKs yield and the dispersion pattern was scrutinized further. The counts of EpASCs, progenitor TA cells, and differentiated / apoptosed cell per se were scored (Fig 2) by plotting the FL1-H value against count of LRKs retaining different amounts of BrdU label; details of numerical workout are described in the legends.

In 42.5ppm iAs exposed group, a notable increase wasseen in counts of LRKs retaining the least amount of BrdU label (see FL1-H 1–3 values in 3D line diagram in Fig 2A); and it was evident in their sum value also (see ∑FL1-H 1–3 values summarized in 100% stacked column diagram in Fig 2B, and the attached respective numerical data sheets). These cells, retaining least BrdU content, represented group of differentiated / apoptosed cells. Count of LRKs, retaining a somewhat greater quantity of BrdU label, showed marginal increase in total yield. This was evident from FL1-H 4–8 values in 3D line diagram in Fig 2A and from the sum values ∑FL1-H 4–8 shown in 100% stacked column diagram in Fig 2B (see also the respective supplement data sheets of Fig 2). These cells represented TA cell (progenitor / unipotent stem cell) population. Counts of LRKs keeping most of BrdU label and representing EpASCsshowed marginal decrease (see FL1-H 9–13 values in 3D line diagram of Fig 2a) as evident also in sum value (∑FL1-H 9–13 values displayed in 100% stacked column diagram in Fig 2b) or the attached numerical data sheet.

A similar effect albeit with greater order of magnitude was observed in 85ppm iAs exposed group; counts of differentiated / apoptosed cells (i.e. LRKs retaining the least amount of BrdU label) increased considerably (Fig 2A and 2B). Counts of TA (progenitor / unipotent stem cells i.e. LRKs retaining comparatively greater amounts of BrdU content) decreased notably. A major loss was observed in the counts of EpASCs retaining the most amount of BrdU label (Fig 2A and 2B). The observed trend of changes at cell count level showed dose responsiveness.

In real meaning, the increase in percent yield of LRKs at 42.5 ppm iAs dose signified a boost in counts of differentiated / apoptosed cells, marginal rise in counts of TA cells, and drop in EpASCs count. Decrease in percent yield of LRKs at 85 ppm iAs dose signified the preponderant loss of TA cell and EpASCs along with considerable increase in differentiated / apoptosed cells. The dose of 85ppm was selected for use in further studies.

### Biological monitoring of transplacental iAs exposure

**Fig 3:**
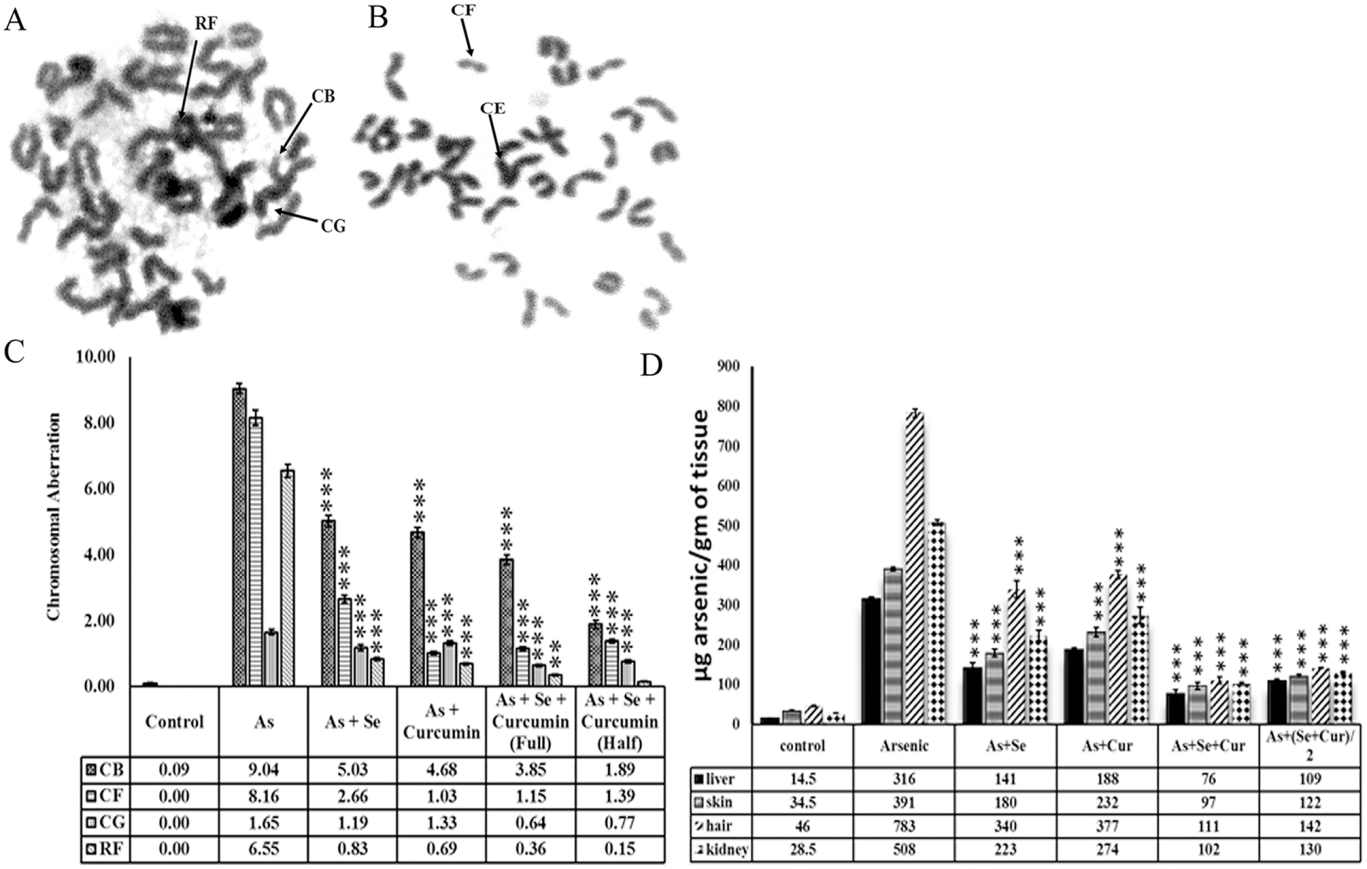
iAs in drinking water induced CA in 0 day old neonate mouse bone marrow cells after 8–18 day *in utero* exposure (A-C) and toxicant burden in hair, skin, liver and kidney of nulliparous female mice after 30 day oral administration (D) and rescue by PO administration of selenium and/or curcumin. Display of RF, CB, CG [A], CF [B], significant changes in formation of CB, CF and RF [C]; data are mean of three different experiments; ± SEM, ‘p’ values are ***<0.001, **<0.01; [D] significant reduction in iAs burden after PO selenium and/or curcumin administration, data are expressed as mean of three different experiments; ± SEM, ‘p’ values are ***<0.001, **<0.01.

Transplacental exposure to iAs was confirmed biologically by monitoring CA in bone marrow cells of zero day old neonates born to mice drinking iAs containing water. The aberrations are shown in Fig 3; these included CB, CG, CF and RF. Alterations were statistically significant (Fig 3A–3C).

The internal exposure to iAs was revealed by the increase of toxicant’s burden in target tissues namely liver, kidney, hair and skin in adult nulliparous female mice (Fig 3D). Mice of all the groups receiving arsenic and / or chemopreventive agents for 30 days showed insignificant changes in body weight (S1b Fig).

### Characterization of transplacental molecular toxicity

**Fig 4:**
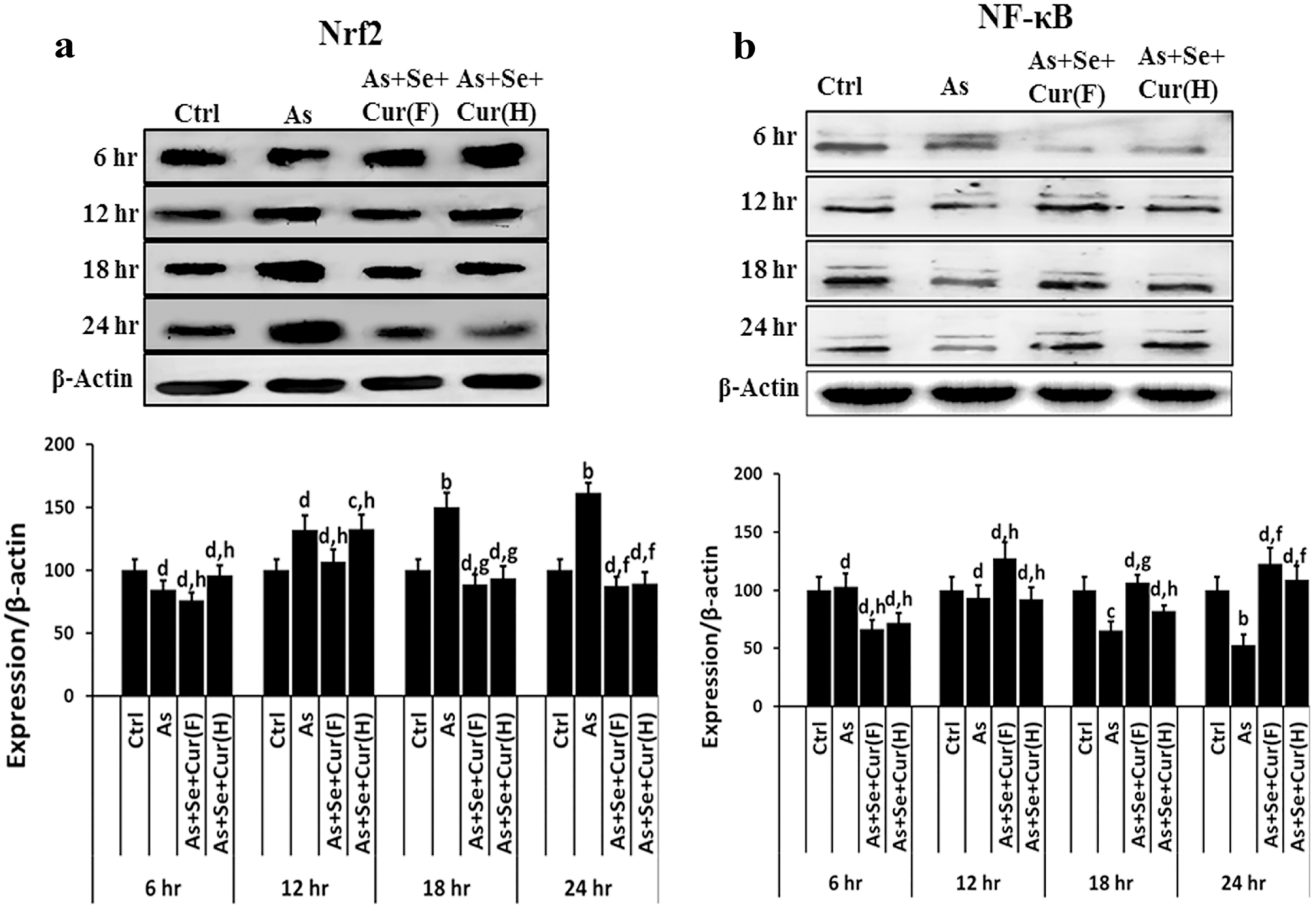
*In utero* exposure to iAs and/or additives induced changes in levels of (a) LRKs biomarkers, and (b) Nrf2 and NF-kB expression in neonate EpASCs. Data are mean of three different experiments; ± SEM, ‘p’ values are ^a^ <0.001, ^b^ <0.01, ^c^ <0.05 and ^d^ >0.05 vs. control and ^e^ <0.001, ^f^ <0.01, ^g^ <0.05 and ^h^ >0.05 vs. arsenic.

Repeated in utero exposure to arsenical drinking water was found to induce alterations in expression of the molecular markers related to EpASCs and the toxicity in neonate epidermis. iAs increased the levels of cytokeratin-10 (differentiated cell marker), TNF-α and PCNA (inflammation and hyper proliferation marker), but decreased the levels of cytokeratin-14 and p63 (the stem cell markers) as summarized in Fig 4A; the effect was dose dependent. The transplacental exposure increased the contents of cytoprotective Nrf2 and pro-inflammatory NFkB also (Fig 4B) revealing the onset of cellular oxidative stress and the subsequent pro-inflammatory molecular change.

**Fig 5:**
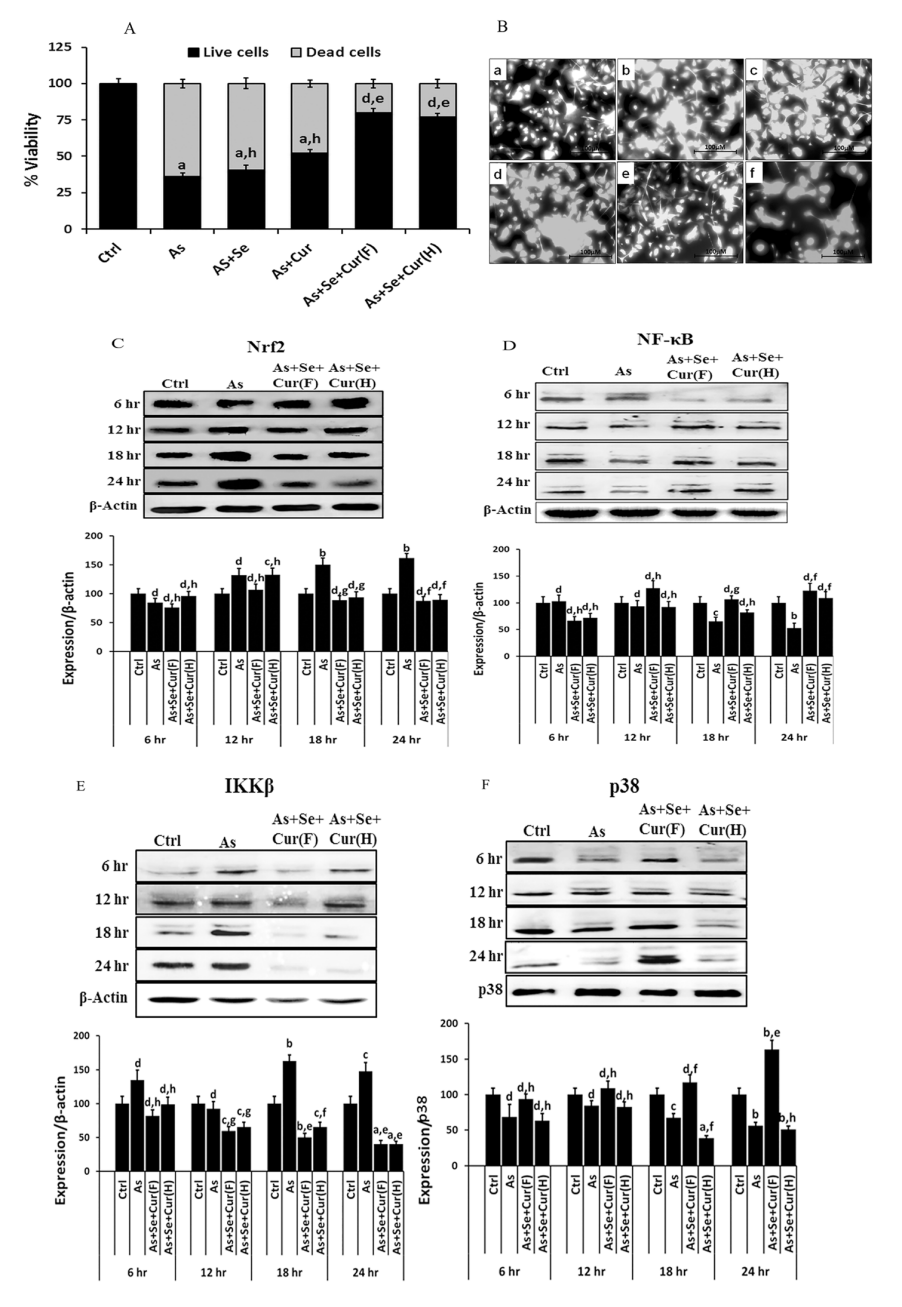
*In vitro* acute exposure to iAs and/or additives induced (A) cytotoxicity and viability, (B) levels of GSH, (C) time course of Nrf2 expression, (D) time course of NFkB expression, (E) time course of IkB expression, (F) time course of phosphorylated-P38 expression in neonate EpASCs. Data are expressed as mean of three different experiments; ± SEM, ‘p’ values are ^a^ <0.001, ^b^ <0.01, ^c^ <0.05,^d^ >0.05 vs. control and ^e^ <0.001, ^f^ <0.01, ^g^ <0.05, ^h^ >0.05 vs. arsenic.

Acute exposure of normal EpASCs to 55μM iAs (the EC_50_ value) resulted in a substantial loss of stem cell viability and the manifestation of cytotoxicity (Fig 5A–5F). Dysregulation in levels of Nrf2, NFkB, IkB protein products and GSH were observed in vitro (Fig 5B–5F). Nrf2 and NFkB protein product level were found to be more. These in vitro observations were in consonance with in vivo findings described earlier in this study. The time course of toxicant induced change is shown in Fig 5B and 5C. The level of Nrf2 protein was found to decrease initially in first 6h of toxicant exposure and to increase thereafter up to 24hrs (Fig 5C). The increase in the Nrf2 protein level was in parallel to in vivo results (see Fig 4b).

Time course study of NFkB expression showed an initial increase in first 6h of toxicant exposure followed by a gradual decrease up to 24hrs (Fig 5D). This observation was a deviation from the in vivo results, and seemed to be a function of possibly different exposure conditions and the test dose. The time course study of IkB expression showed dysregulation after iAs exposure, and was found to be complimentary to the expression pattern of NFkB. As evident in Fig 5E, after initial decrease in first 6h, IkB expression increased linearly up to 24hrs.

iAs inhibited the phosphorylation of MAPK family member p38 in stem cells (Fig 5F); however, retention of partial activity was observed for next 24hrs. The attenuation of p38-phosphorylation indicated inhibition of redox regulated MAPK in stem cells and validated the stress inducing potential of iAs. It was in line with the observed increase in the counts of differentiating cell populations in vitro.

### Rescue of iAs induced disorder in LRKs counts and changes at molecular level

Co-administration of selenite and curcumin combine countered iAs induced transplacental changes both at cellular and molecular level; the preventive effect was evident both in vivo (Fig 1D–1G) and in vitro (Fig 5A–5F). The EC_50_ valuefor arsenite, selenite, and curcumin were determined for use in in-vitro experiments and are summarized in S1 Fig. At the cellular level, selenite and curcumin counteracted the changes in counts of EpASCs and progenitor cells in vivo. The preventive agents restricted the decrease in EpASCs count by approximately 50% compared to the decrease observed in their absence (Fig 2A and 2B). Interestingly, selenite and curcumin combine hampered the toxicant induced increase in formation of differentiated and progenitor TA cells. It was reflected in counts of differentiated / apoptosed and TA cells as shown in the numerical datasheet (Fig 2A and 2B). Individually, selenite or curcumin rescued the loss in counts of EpASCs counts inefficiently (Fig 2A).

At the molecular level, selenite and curcumin foiled iAs induced changes in content of the cytoprotective Nrf2, antioxidant GSH, inflammation mediator NFkB in adult stem cell (Fig 4B). Selenium and curcumin combine was comparatively more efficient than each individually to prevent the iAs induced descend in adult stem cell viability, ascend in CMFDA-reactive GSH content, and change in expression of the toxicity markers (Fig 5A–5F). The time course of molecular changes in EpASCs was prevented to near normal values by selenite and curcumin combine.

The additives counterbalanced other features of iAs toxicity as well, namely (i) increase in CA in bone marrow cells (Fig 3A–3C); (ii) the toxicant burden in tissues (Fig 3D) (iii) increase in de novo synthesis of Nrf2, NFkB in vivo (Fig 4B); and (iv) the cytotoxicity, the altered biochemical parameters like increase in IkB expression and p38 phosphorylation, and the decrease in expression of Nrf2, NFkB, IkB and GSH biosynthesis in vitro (Fig 5B–5F).

A strong induction in the levels of CMFDA-reactive GSH was seen in stem cells after exposure to iAs (Fig 5B). A similar response was seen again after exposure to a combination of selenite and iAs (Fig 5C) or curcumin and iAs (Fig 5D). These observations vindicated the finding of iAs induced NFkB, IkB, Nrf2 gene over expressions. Induction in levels of CMFDA-reactive GSH in stem cells might however be confounded by the delay in repression of demand-driven GSH biosynthesis.

In essence, the combination of selenite and curcuminproved to be comparatively more effective for regulation of the iAs induced increase in intracellular levels of GSH in adult stem cells to near-normal values (Fig 5B–5F) in addition to increase in (a) expressions of NFkB, p38 (Fig 5D and 5F) and (b) formation of TA cells (Fig 2A and 2B).

### *In silico* study of molecular interactions between curcumin, selenite and Keap1

Results are summarized in S1 & S2 Tables. Individually,curcumin and GS-AsH-SG (the stable metabolite of arsenite) were found to interact with Keap-1 registering a higher PatchDock Score of 5022 and 5174 respectively. GS-Se-SG, the stable metabolite of selenite, also interacted with Keap-1 however on a relatively low score of 4858 (S1 Table).

Investigations revealed that after binding with curcumin (S2a Fig), ZDock score of Keap-1 decreased from 11.68 to 8.58 (S2 Table) suggesting relatively better potential of curcumin-Keap-1 complex for interacting with Nrf2 and activating it as well. Ligand Keap-1 was found to interact with natural receptor Nrf2 (S2d Fig) normally with a ZDock Score of 11.68 (S2 Table).

## Discussion

This study demonstrated that repeated in utero exposure to arsenical drinking water influenced adult stem cell homeostasis in neonate epidermis at the time of birth by inducing a significant change in counts of EpASCs, unipotent / progenitor TA cells, and differentiated cells along with the alterations in levels of Nrf2, NFkB, IkB, TNF-α protein products and GSH. The results signified the acquisition of disorder in neonate EpASCs homeostasis in vivo after in utero exposure to iAs in drinking water. The ultimate foci of damage were the counts of adult stem cells and progenitor cells, though in different proportions and dimensions.

The mechanism of aberration in EpASCscounts seemed to couple with a loss in stemness and with a surge of differentiation. These observations are in line with hazardous effects of iAs described in literature since long [30]. Changes in skin with respect to stem cell homeostasis disorder, such as hyperkeratosis, acanthosis are still being reported both in vivo in humans [31] and in vitro in human skin equivalent system [32]. The increase in incidences of an array of adverse health effects as well as rise in mortality (from cancer and cardiorespiratory diseases) are described both in childhood and adulthood in populations transplacently exposed to iAs [11–15,33–40]. Early life exposure to arsenic and the resultant acquisition of acute / long-term impairment in lung function and tissue mechanics, postnatal development, and behavioural changes is reported in laboratory animals also. The gaining of predisposition to disease is explained also by gene expression manipulations triggered by transplacental iAs exposure [41–44]. Chronic exposure to iAs is inevitable in the early part of life due to contamination of the food chain beyond permissible limit whether in drinking water and/or staple food worldwide [45–47].

The trigger of change in EpASCs homeostasis is accompanied with oxidant stress and resultant inflammation mediators via over-expression of rapidly acting and resident transcription factor Nrf2 [48–51] plus pro-inflammatory molecules TNF-α, NFkB [49]. The activation of the Nrf2-Keap1 antioxidant pathway by transplacental iAs exposure has been reported in humans as reviewed recently [49–50]; nevertheless, there is a paucity of information in the literature on this issue in adult stem cell. The present study provides this evidence for the first time.

This study further demonstrated the role of cytokine regulated inflammation and MAPK pathway in the mechanism of action of iAs in adult stem cells. Both the toxicant stress and the related biochemical changes seemed to trigger ultimately the de novo biosynthesis of GSH and other antioxidant molecules in stem cells as evident from the data on CMFDA-reactive GSH levels. p38 kinase is a part of MAPK that is activated by dual kinases (MKKS), and responds to extracellular stress stimuli for cell differentiation and apoptosis. Although iAs partially inhibited p38 phosphorylation in our study, the remnant activity of phosphorylated p38 seemed to support the surge of differentiation, the inconsistencies in counts of EpASCs and progenitor cells, and the ensuing paradigm shift in dynamics of homeostasis in vivo [52–54].

EpASCs homeostasis disorder may impair vital functions like wound repair in tissues, which eventually could render vulnerability to diseases and disorders of multiple organ systems in humans. The hypothesis on persistent suboptimal counts of EpASCs after prolonged in utero exposure to iAs could plausibly be applicable in other ectoderm tissues as well. However, more studies are required to strengthen the extension of this hypothesis to other tissues.

**Fig 6:**
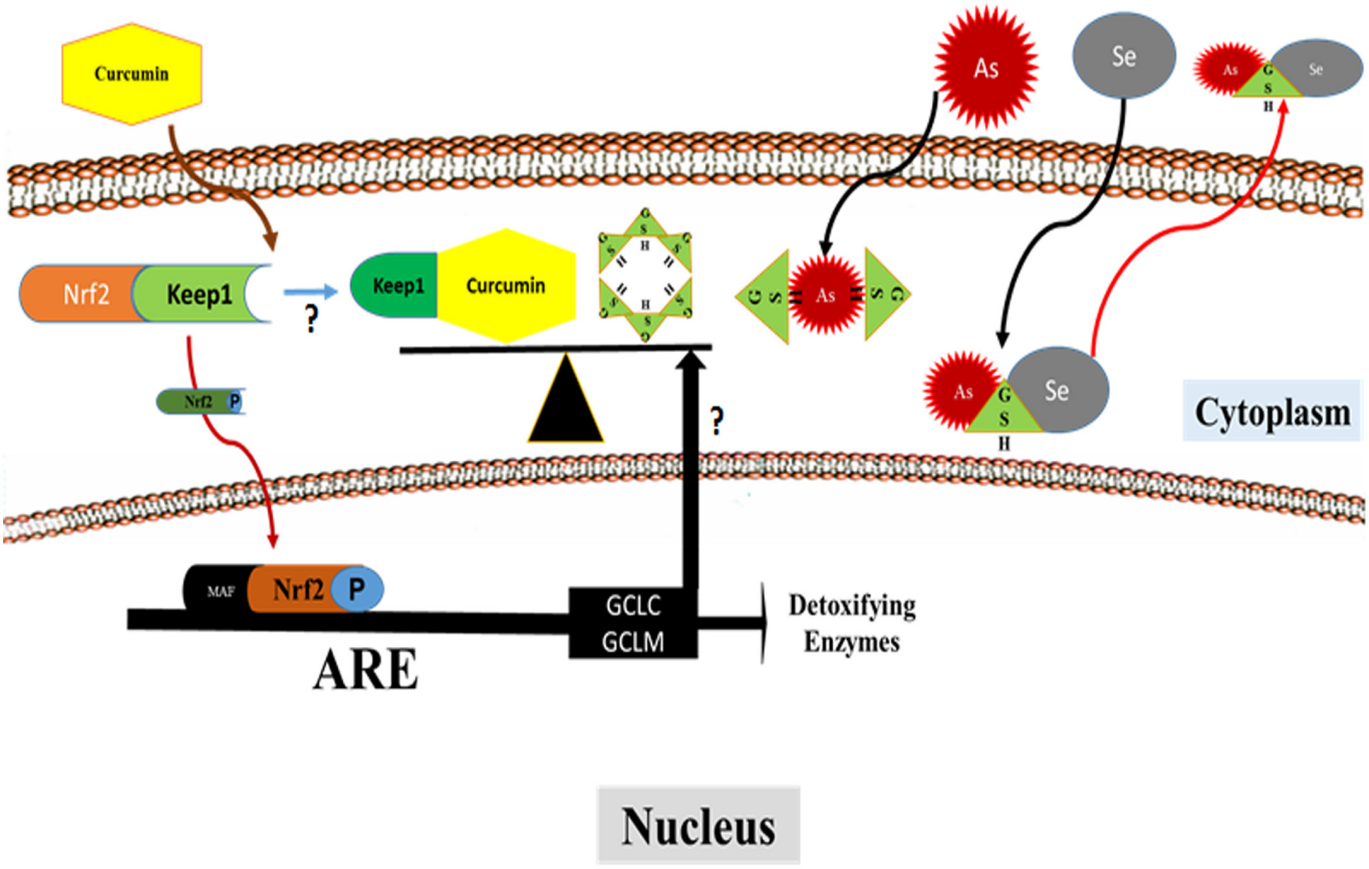
Pathway diagram showing Nrf2 activation and enhanced GSH biosynthesis through curcumin and release of arsenic via selenium and GSH complex.

In current study, the stem cell homeostatic disorder and the experimental aesenicosis were effectively countered by repeated simultaneous intake of combined selenite and curcuminin utero. The rescue seemed to operate through de novo GSH biosynthesis triggered both by oxidant stress and Nrf2 & NFkB over expression (Fig 6).

Data on expressions of NFkB, IkB, Nrf2, and CMFDA-reactive GSH in iAs exposed EpASCs both in vivo and in vitro corroborates this view and also vindicates the in silico prediction for (i) Nrf2 release by curcumin, (ii) the subsequent de novo GSH biosynthesis, and (iii) the change in status of oxidative stress. The in silico study has allowed an insight into the contribution of arsenite, selenite, and curcumin in activation of Nrf2 for inducing de novo GSH biosynthesis. In silico although arsenite, selenite, curcumin made complex with Keap-1 (the natural ligand for Nrf2) as illustrated in S2a–S2c Fig and S1 & S2 Tables, curcumin-Keap-1 complex, however, emerged to be a more potent ligand for Nrf2 than the natural ligand Keap-1 alone (S2d Fig, S1 & S2 Tables); it appeared to spoil the affinity of Keap-1 alone for Nrf2. These possibilities suggested the contribution of curcumin to induce the de novo GSH biosynthesis via cytoprotective Nrf2 activation in addition to the pro-inflammatory NFkB pathway. Arsenite and selenite contribute to augmentation in de novo biosynthesis of GSH through over-expression of both Nrf2 as well as NFkB. These observations corroborate with the findings of CMFDA-reactive GSH status in EpASCs. The other parallel contributes to prevention of stem cell homeostasis disorder could be the (i) rapid mobilization of accumulated iAs from hair, skin, liver attributable to its increased metabolism and disposal in GSH enriched cells finally reducing the toxic burden in tissues, and (ii) prevention of iAs induced DNA damage and loss of cell viability. These observations may help explain the wound healing property of curcumin observed traditionally as well as described in the literature [55,56].

The present study provides a lead for experimental in utero chemoprevention of transplacental iAs toxicity using essential micronutrient and food additives and regenerative-naturopathy in infants born to iAs-exposed mothers that are presumably predisposed to ailments, diseases, and disorders developing later in adulthood. The bio-remedial measures, available in the literature, are somatic cell specific and are transient. Engineering the removal of arsenic from potable water is a Herculean task, besides being cost-intensive, toxic sludge waste generating, and failing the challenge of removing metalloids from food chain. The use of essential micronutrient and dietary supplements to contain and / or remove iAs-induced disorders in stem cell homeostasis and to detoxify iAs exposed subjects could be a potentially effective, logical, non-toxic, and health-improving strategy.

## Conclusion

This study evidently demonstrated the acquisition of adult stem cell homeostasis disorder and critical molecular changes after chronic in utero exposure to iAs early in life and their efficient chemoprevention by selenite and curcumin combine. Manipulation in counts of EpASCs and the unipotent / progenitor TA cells, and the differentiated cells in the neonate epidermis at the beginning of neonatal age is hypothesized to be detrimental to the functions of skin and form a cellular basis to vulnerability for arsenicosis later in life. After in utero iAs exposures, the adult stem cell loss, accumulation of stem cell count aberrations, and homeostasis disorder together with continual insufficient antioxidant activity and inadequate toxicant disposition can possibly be the crucial contribute to the increases in disease susceptibility and disease burden observed in neonatal and adulthood life. In silico studies support the observed chemopreventive potential of the studied food additives to activate Nrf2-Keap1 dependent regeneration of endogenous antioxidant GSH for the rescue of iAs impaired EpASCs homeostasis.

## Supporting Information

S1 Fig EC_50_value of arsenic, selenite, curcumin in epidermal adult stem cell using cell viability assay (MTT assay) (Fig A). Body weight of mice following 30 day exposure to arsenic, arsenic with selenium, arsenic with curcumin, arsenic with selenium and curcumin, and arsenic with selenium and curcumin in half dose (Fig B).
(DOCX)

S2 Fig Molecular Interaction Analysis of Keap1-Curcumin (Figure Generated by Discovery Visualizer) (Fig A). Molecular Interaction Analysis of Keap1-GS-AsH-SG, (Figure Generated by Discovery Visualizer) (Fig B). Molecular Interaction Analysis of Keap1-GS-Se-SG, (Figure Generated by Discovery Visualizer) (Fig C). Molecular Interaction Analysis of Keap1 & Nrf2 (Figure Generated by Discovery Visualizer) (Fig D)
(DOCX)

S1 Table Molecular Interaction Analysis of Keap-1 with Curcumin, GS-AsH-SG & GS-Se-SG using PatchDock Server and Discovery Visualizer. (DOCX)

S2 Table Molecular Interaction study of Keap1, Keap1-Curcumin, Keap1-[GSH-Se-GSH], and Keap1-[GS-AsH-SG] with Nrf2 using ZDOCK. S2 Table Footnote. In column “No. of Hydrogen Bond”- A, B and UNK indicates aminoacid residues of Keap1, Nrf2 & Curcumin, [GS-Se-SG], [GS-AsH-SG] respectively.
(DOCX)

## Abbreviations

AAS: Atomic Absorption Spectrophotometer
EpASCs: epidermis adult stem cells
BrdU: 5-bromo-2'-deoxyuridine
CA: chromosomal aberrations
CB: chromosomal breaks
CF: chromosomal fragment
CG: chromosomal gap
CK10: cytokeratin 10
CK14: cytokeratin 14
CMFDA: 5-chloromethylfluorescein di-acetate
CPCSEA: Committee for the Purpose of Control and Supervision on Experiments on Animals
DMSO: dimethyl sulfoxide
EpASCs: Epidermal adult stem cells
GS-AsH-SG: arseno-diglutathione complex
GS-Se-SG: seleno-diglutathione complex
iAs: inorganic Arsenic
IkB: Inhibitor of NF-κB activity
Keap1: Kelch like-ECH-associated protein 1
LRKs: label (BrdU)-retaining keratinocytes
MAPK: Mitogen activated protein kinase
MTT: 3-(4, 5-dimethylthiazol-2-yl)-2, 5-diphenyl tetrazolium bromide
NFkB: nuclear factor kappa-light-chain-enhancer of activated B cells
Nrf2: Nuclear factor (erythroid-derived 2)-like 2
PO: per os
PCNA: proliferating cell nuclear antigen
RF: ring formation
TA: transiently amplifying
TNF-α: tumor necrosis factor alpha
