# Correction: Disruption of Skin Stem Cell Homeostasis following Transplacental Arsenicosis; Alleviation by Combined Intake of Selenium and Curcumin

**DOI:** 10.1371/journal.pone.0146001

**Published:** 2015-12-30

**Authors:** Shiv Poojan, Sushil Kumar, Vikas Verma, Anupam Dhasmana, Mohtashim Lohani, Mukesh K. Verma

The image for Fig 4 is incorrect. Please view the correct Fig 4 here.

**Fig 4:**
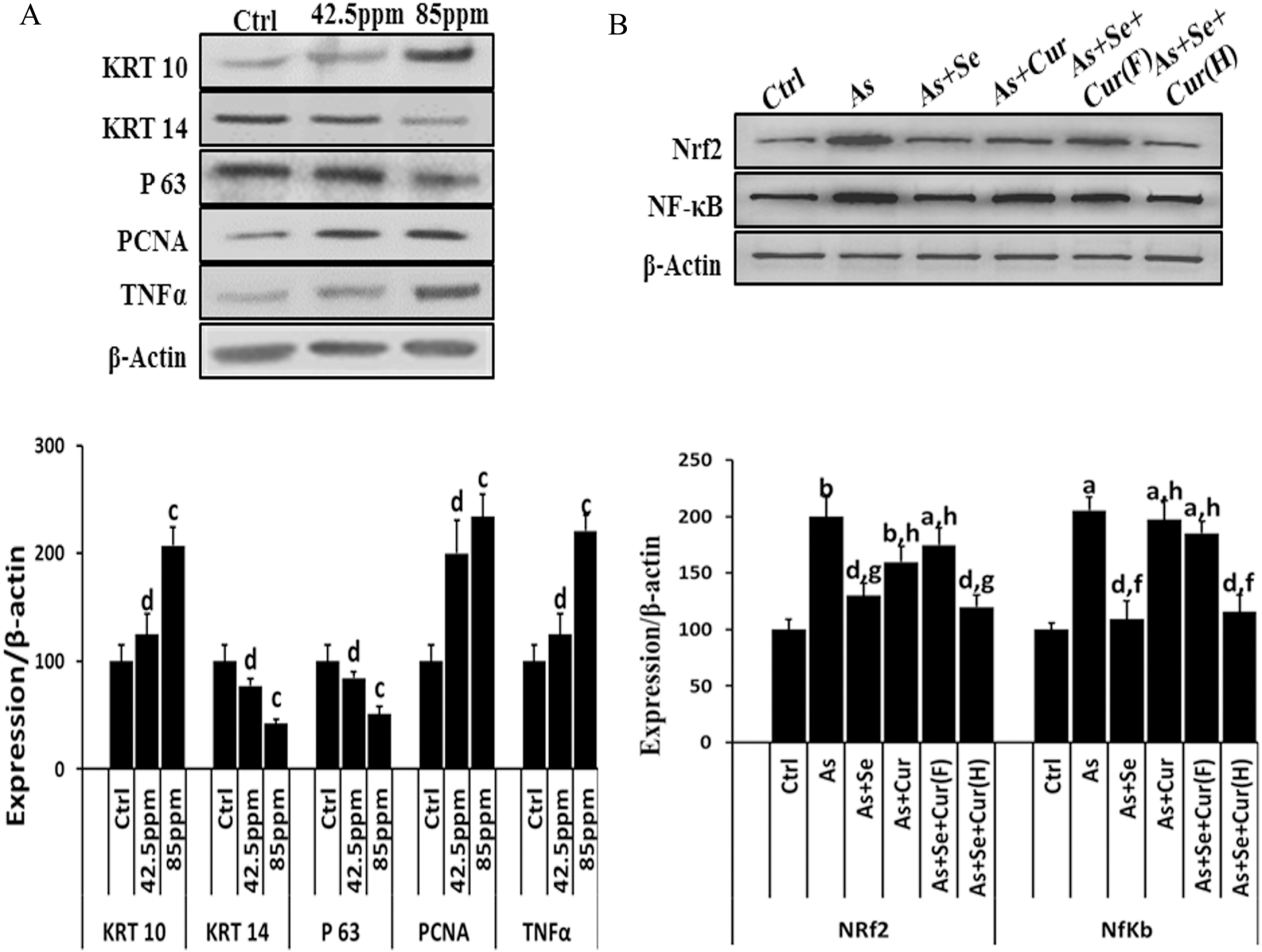
*In utero* exposure to iAs and/or additives induced changes in levels of (A) LRKs biomarkers, and (B) Nrf2 and NF-kB expression in neonate EpASCs. Data are mean of three different experiments; ± SEM, ‘p’ values are ^a^ <0.001, ^b^ <0.01, ^c^ <0.05 and ^d^ >0.05 vs. control and ^e^ <0.001, ^f^ <0.01, ^g^ <0.05 and ^h^ >0.05 vs. arsenic.

## References

[1] Poojan S, Kumar S, Verma V, Dhasmana A, Lohani M, Verma MK (2015) Disruption of Skin Stem Cell Homeostasis following Transplacental Arsenicosis; Alleviation by Combined Intake of Selenium and Curcumin. PLoS ONE 10(12): e0142818 doi: 10.1371/journal.pone.0142818 26624291 10.1371/journal.pone.0142818 PMC4666640

