# Supplementary figures and images for: Disruption of Skin Stem Cell Homeostasis following Transplacental Arsenicosis; Alleviation by Combined Intake of Selenium and Curcumin
Source: PLoS One. 2015 Dec 1;10(12):e0142818. doi: 10.1371/journal.pone.0142818 (PMC4666640; doi:10.1371/journal.pone.0142818)

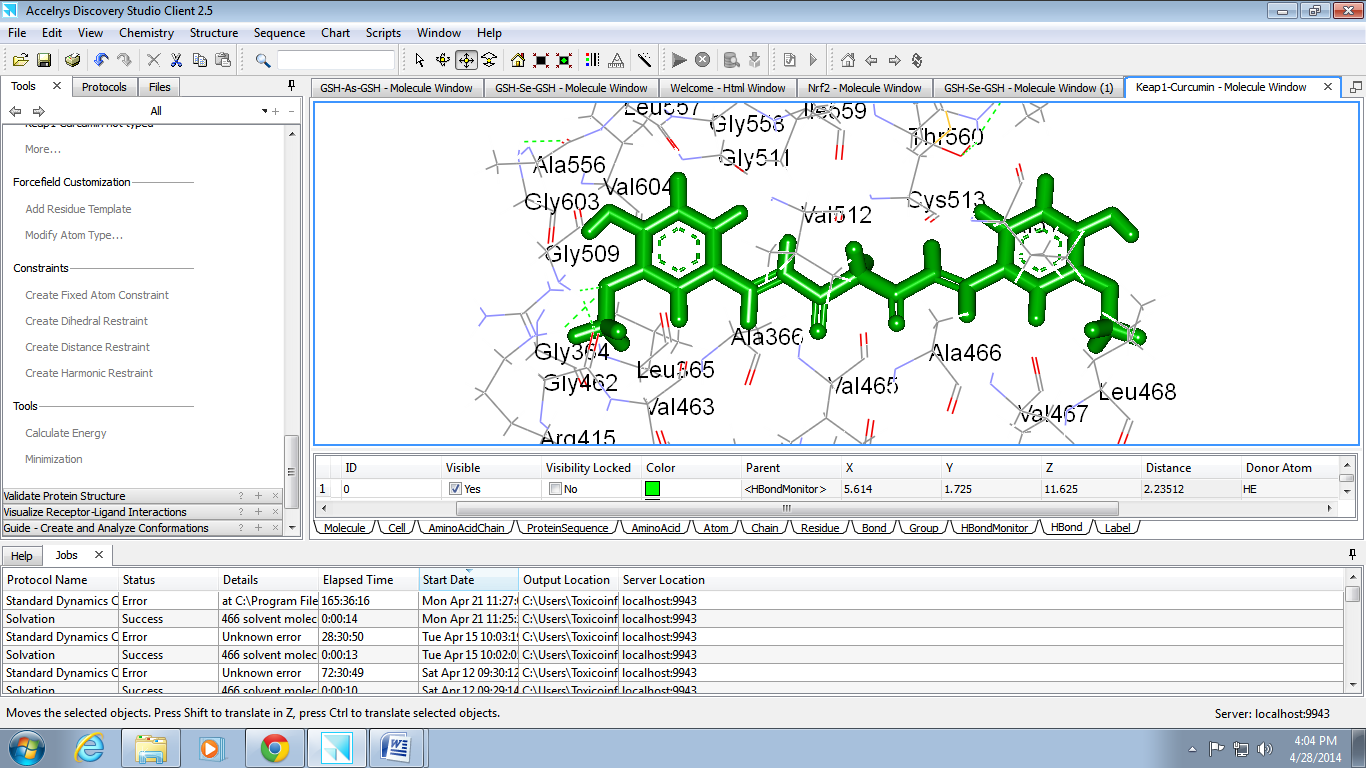


**Fig. S2A**

**
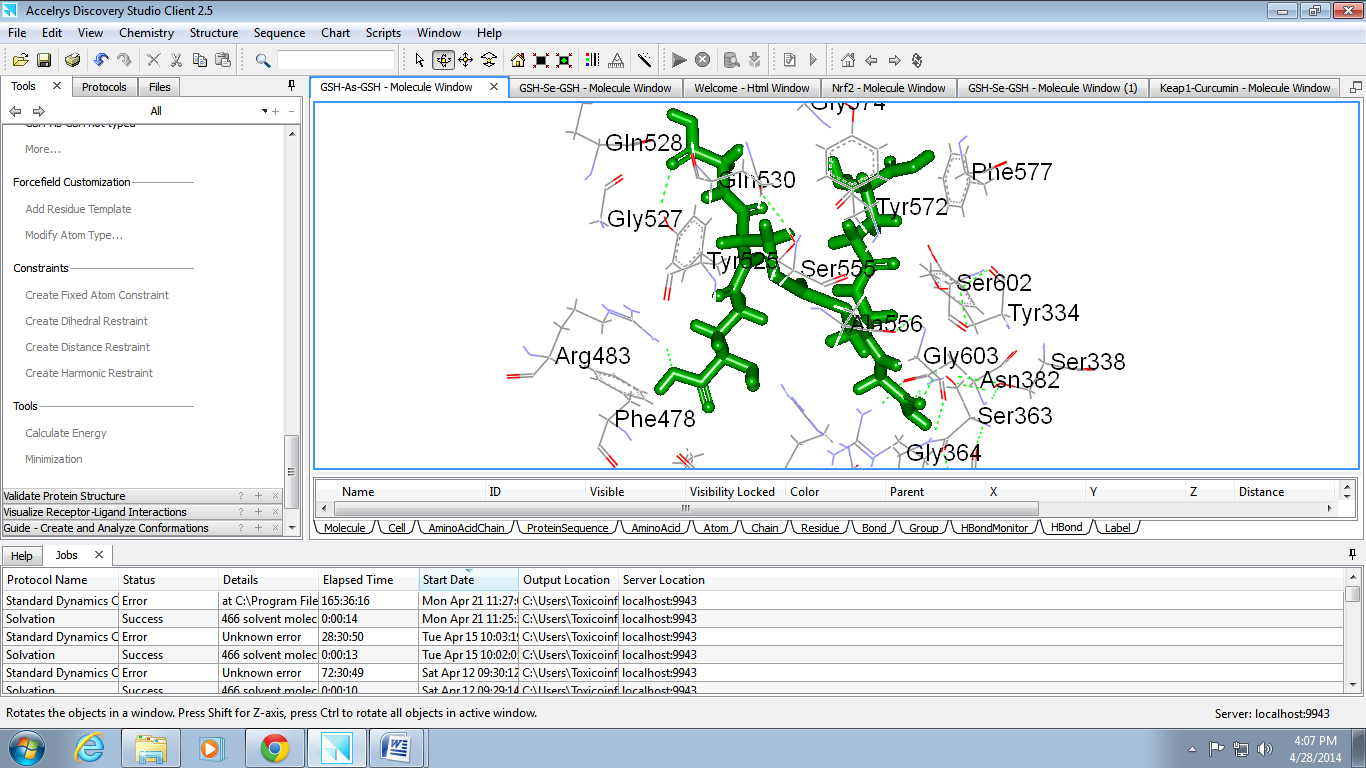
**

**Fig. S2B**


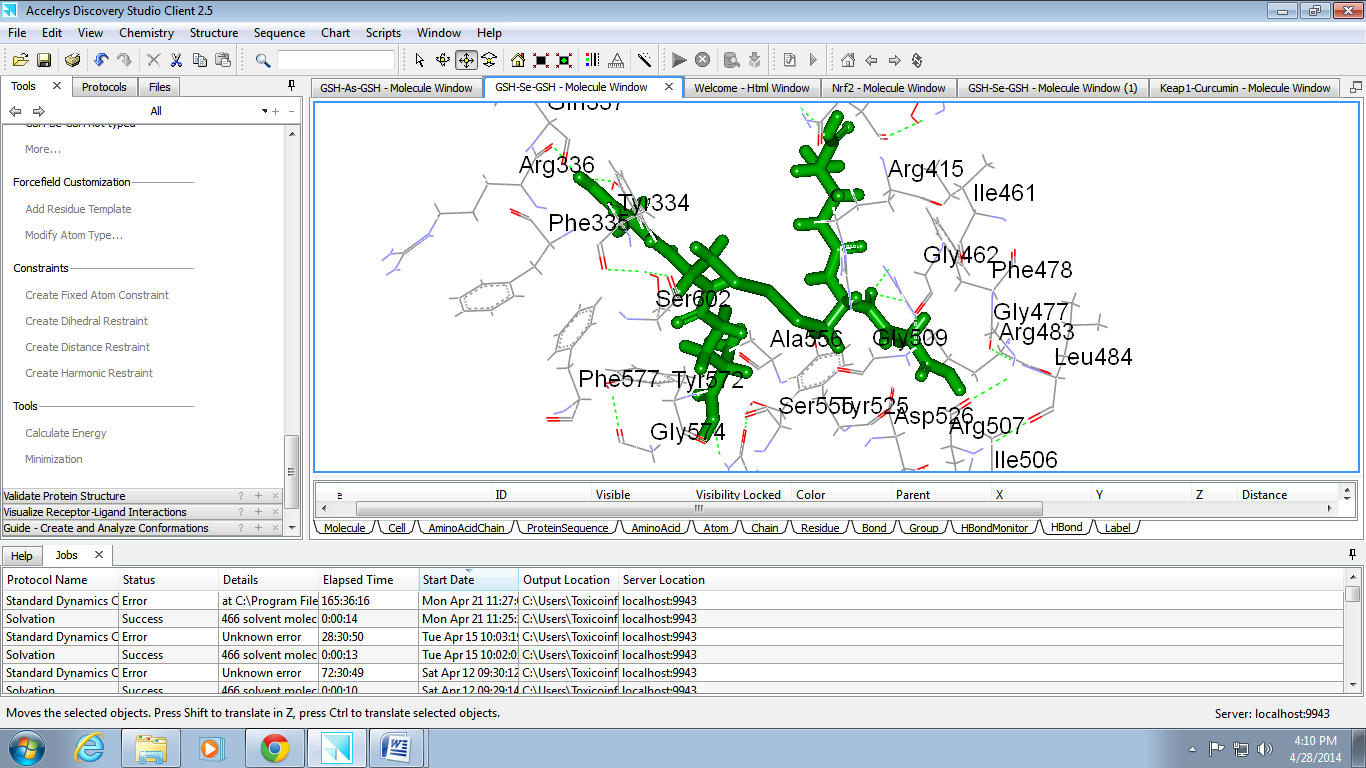


**Fig. S2C**

**
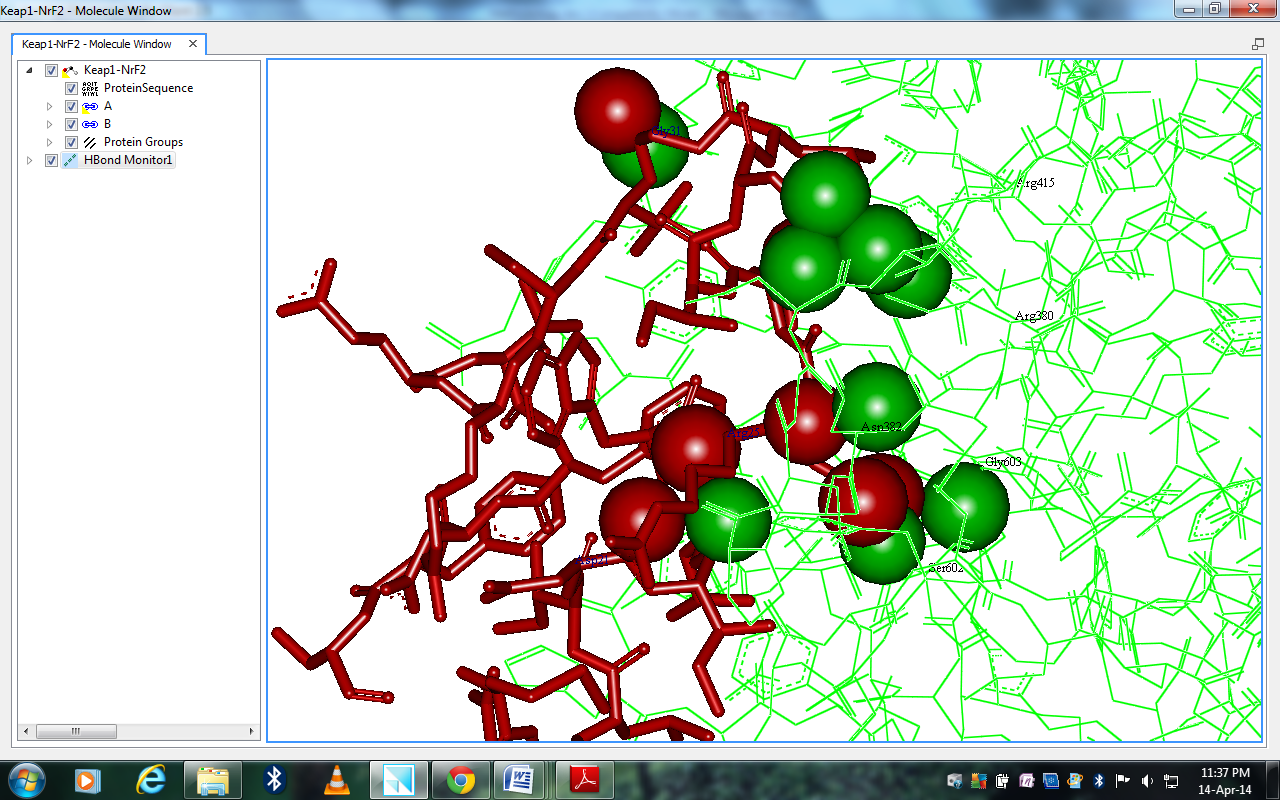
**

**Fig. S2D**

Supplement: S2 Fig — Molecular Interaction Analysis of Keap1-GS-AsH-SG, (Figure Generated by Discovery Visualizer) (Fig B). Molecular Interaction Analysis of Keap1-GS-Se-SG, (Figure Generated by Discovery Visualizer) (Fig C). Molecular Interaction Analysis of Keap1 & Nrf2 (Figure Generated by Discovery Visualizer) (Fig D) (DOCX) [file pone.0142818.s002.docx]
