# Supplementary material for: Disruption of Skin Stem Cell Homeostasis following Transplacental Arsenicosis; Alleviation by Combined Intake of Selenium and Curcumin
Source: PLoS One. 2015 Dec 1;10(12):e0142818. doi: 10.1371/journal.pone.0142818 (PMC4666640; doi:10.1371/journal.pone.0142818)
Supplement: S1 Table — (DOCX) [file pone.0142818.s003.docx]

**Table S1.**

| **S.**  **No** | **Receptor** | **Ligand** | **PatchDock**  **Score** | **Residues** | **H-Bond Residue** | **H-Bond Distance** |
| --- | --- | --- | --- | --- | --- | --- |
| 1 | Keap1 | Curcumin | 5022 | Gly364,Leu365  Ala366,Arg415  Ile416,Gly417  Val418,Gly462  Val463,Val465  Ala466,Val467  Leu468,Gly509  Gly511,Val512  Cys513,Val514  Ala556,Leu557  Gly558,Ile559  Thr560,Val561  Gly603,Val604 | 1)A:ARG415:HE - :UNK1:O2  2)A:ARG415:HH21 - :UNK1:O2 | 1) 2.23512  2)1.97525 |
| 2 | Keap1 | GS-AsH-SG | 5174 | Tyr334,Ser338  Ser363,Gly364  Arg380,Asn382  Asn414,Arg415  Ile461,Phe478  Arg483,Tyr525  Gly527,Gln528  Gln530,Ser555  Ala556,Tyr572  Gly574,Phe577  Ser602,Gly603 | 1)A:SER363:HG - :UNK1:O30  2)A:ASN382:HD22 - :UNK1:O30  3)A:ASN382:HD22 - :UNK1:O31  4)A:ARG483:HH12 - :UNK1:O45  5)A:TYR525:HH - :UNK1:O37 | 1)1.81097  2)2.28204  3)1.17347  4)1.37978  5)2.44511 |
| 3 | Keap1 | GSH-Se-GSH | 4858 | Tyr334,Phe335  Arg336,Gln337  Arg380,Asp389  Asn414,Arg415  Ser431,Ile461  Gly462,Gly477  Phe478,Arg483  Leu484,Ile506  Arg507,Gly509  Tyr525,Asp526  Gln530,Ser555  Ala556,Tyr572  Gly574,Phe577 | 1):UNK1:H62 - A:ARG336:O  2):UNK1:H62 - A:TYR334:OH  3)A:ARG483:HH12 - :UNK1:O32  4)A:ARG483:HH11 - :UNK1:O32  5)A:ARG415:HH11 - :UNK1:O1  6)A:ASN414:HD22 - :UNK1:O45  7)A:ASN414:HD21 - :UNK1:O45  8)A:TYR334:HH - :UNK1:O30  9)A:TYR334:HH - :UNK1:O29  10)A:GLN530:HE22 - :UNK1:O23 | 1)2.30238  2)1.40602  3)2.42698  4)1.85304  5)2.00602  6)1.86327  7)1.87898  8)2.11858  9)2.46461  10)1.31755 |
