# Supplementary material for: Disruption of Skin Stem Cell Homeostasis following Transplacental Arsenicosis; Alleviation by Combined Intake of Selenium and Curcumin
Source: PLoS One. 2015 Dec 1;10(12):e0142818. doi: 10.1371/journal.pone.0142818 (PMC4666640; doi:10.1371/journal.pone.0142818)
Supplement: S2 Table — In column “No. of Hydrogen Bond”- A, B and UNK indicates aminoacid residues of Keap1, Nrf2 & Curcumin, [GS-Se-SG], [GS-AsH-SG] respectively. (DOCX) [file pone.0142818.s004.docx]

**Table S2.**

| **S. No** | **Receptor** | **Ligand** | **ZDOCK Score** | **ZDOCK**  **Rank** | **No. of Hydrogen Bond** |
| --- | --- | --- | --- | --- | --- |
| 1 | Keap1 | NrF2 | 11.68 | -92.029 | A:TYR334:OH - B:ASP21:O  A:TYR334:OH - B:ILE22:O  A:ARG380:NE - B:ASP29:OD1  A:ARG380:NE - B:ASP29:OD2  A:ARG380:NH1-B:ASP29:OD1  A:ARG380:NH1 - B:ASP29:OD2  A:ARG380:NH2-B:ASP29:OD  A:ARG380:NH2 - B:ASP29:OD2  A:ASN382:ND2 - B:ARG25:O  A:ARG415:NH1 - B:ASP27:OD1  A:ARG483:NH2 - B:GLY31:O  A:SER602:OG - B:GLN26:OE1  A:GLY603:N - B:GLN26:OE1  B:ARG25:N - A:TYR334:OH  B:GLN26:NE2 - A:SER602:OG |
| 2 | Keap1-Curcumine | NrF2 | 8.58 | -88.823 | A:TYR334:OH - B:ASP21:O  A:TYR334:OH - B:ILE22:O  A:ARG380:NE - B:ASP29:OD1  A:ARG380:NE - B:ASP29:OD2  A:ARG380:NH1 - B:ASP29:OD1  A:ARG380:NH1 - B:ASP29:OD2  A:ARG380:NH2 - B:ASP29:OD1  A:ARG380:NH2 - B:ASP29:OD2  A:ASN382:ND2 - B:ARG25:O  A:ARG415:NE - :UNK1:O2  A:ARG415:NH1 - B:ASP27:OD1  A:ARG415:NH2 - :UNK1:O2  A:ARG483:NH2 - B:GLY31:O  A:SER602:OG - B:GLN26:OE1  A:GLY603:N - B:GLN26:OE1  :UNK1:O27 - A:ALA556:O  B:ARG25:N - A:TYR334:OH  B:GLN26:NE2 - A:SER602:OG  A:TYR334:OH - B:ASP21:O  A:TYR334:OH - B:ILE22:O  A:ARG380:NE - B:ASP29:OD1  A:ARG380:NE - B:ASP29:OD2  A:ARG380:NH1 - B:ASP29:OD1  A:ARG380:NH1 - B:ASP29:OD2  A:ARG380:NH2 - B:ASP29:OD1  A:ARG380:NH2 - B:ASP29:OD2  A:ASN382:ND2 - B:ARG25:O  A:ARG415:NE - :UNK1:O2  A:ARG415:NH1 - B:ASP27:OD1  A:ARG415:NH2 - :UNK1:O2  A:ARG483:NH2 - B:GLY31:O  A:SER602:OG - B:GLN26:OE1  A:GLY603:N - B:GLN26:OE1  :UNK1:O27 - A:ALA556:O  B:ARG25:N - A:TYR334:OH  B:GLN26:NE2 - A:SER602:OG |
| 3 | Keap1-[GS-Se-SG] | NrF2 | 11.56 | -90.660 | A:TYR334:OH - :UNK1:O29  A:TYR334:OH - :UNK1:O30  A:ARG336:NE - B:PHE39:O  A:ARG336:NH2 - B:PHE39:O  A:ARG380:NH1 -B:GLN26:OE1  A:ARG380:NH2 -B:GLN26:OE1  A:ASN414:ND2 - :UNK1:O45  A:ARG415:NH1 - :UNK1:O1  A:ARG483:NH1 - :UNK1:O32  A:ARG483:NH2 - B:LEU30:O  A:TYR525:OH - B:GLY31:O  A:GLN530:NE2 - :UNK1:O23  :UNK1:O23 - A:GLN530:OE1  :UNK1:N26 - A:TYR334:OH  :UNK1:O30 - A:TYR334:OH  :UNK1:O30 - A:ARG336:O  :UNK1:O30 - B:PHE37:O  :UNK1:O37 - A:GLY477:O  :UNK1:N42 - B:ASP27:OD1 |
| 4 | Keap1-[GS-AsH-SG] | NrF2 | 11.60 | -90.951 | A:SER363:OG - :UNK1:O30  A:ASN382:ND2 - :UNK1:O30  A:ASN382:ND2 - :UNK1:O31  A:THR481:OG1 - B:LEU30:O  A:ARG483:NH1 - :UNK1:O45  A:SER555:OG - :UNK1:S7  A:SER602:OG - :UNK1:O16  :UNK1:O31 - A:ASN382:OD1  :UNK1:O38 - B:VAL36:O  :UNK1:O38 - B:ASP38:O  :UNK1:O45 - B:ASP27:OD1  :UNK1:O45 - B:ASP27:OD2 |
